# Therapeutic Effect of Polidocanol Sclerotherapy on Oral Vascular Malformations

**DOI:** 10.3390/dj9100119

**Published:** 2021-10-14

**Authors:** Satoshi Fukuzawa, Kenji Yamagata, Makiko Okubo-Sato, Kazuhiro Terada, Fumihiko Uchida, Naomi Ishibashi-Kanno, Hiroki Bukawa

**Affiliations:** Department of Oral and Maxillofacial Surgery, Institute of Clinical Medicine, Faculty of Medicine, University of Tsukuba, 1-1-1 Tennodai, Tsukuba 305-8575, Ibaraki, Japan; y-kenji@md.tsukuba.ac.jp (K.Y.); mokubo0221@gmail.com (M.O.-S.); mask.dis.71r@gmail.com (K.T.); uchiyamada1031@yahoo.co.jp (F.U.); greened_amethyst829@hotmail.com (N.I.-K.); bukawa-cuh@umin.ac.jp (H.B.)

**Keywords:** oral vascular malformation (VM), polidocanol, sclerotherapy

## Abstract

Various treatments for oral vascular malformation (VM) have been reported. Polidocanol and absolute ethanol have also been reported for sclerotherapy. However, there are still few reports on the therapeutic effect and dosage of polidocanol sclerotherapy. Therefore, we examined its therapeutic effects on oral VM. There were 17 sites of VMs, with nine patients diagnosed with oral VM at the Department of Dental and Oral Surgery, Tsukuba University Hospital. The medical records were retrospectively investigated to determine the site, hemangioma volume, polidocanol injection volume, and therapeutic effect. The volume of hemangiomas was calculated using magnetic resonance images. Based on the site, oral VMs were observed in the tongue, buccal mucosa, lips, and oral floor in eight, three, five, and one patients, respectively. The average size of the site was 3071 mm^3^. The average injection dose of polidocanol at one site was 2.86 mL, the average number of administrations was 1.6, and the response rate was 88.2%. No adverse events were observed. The median numerical rating scale scores were 2/10 (0–6/10) and 0/10 (0–1/10) the day after surgery and 1 week after surgery, respectively. Univariate regression analysis of the total dose in successful cases provided the following formula: 1.3 + 0.00025 × volume (mm^3^) (mg). Polidocanol sclerotherapy is an effective treatment method for oral VM.

## 1. Introduction

Hemangiomas occur frequently in the head and neck region, especially in the tongue and buccal mucosa. Mulliken et al. divided hemangiomas and vascular malformations (VMs) according to clinical conditions and endothelial cell characteristics [1]. Mulliken et al. and Jackson et al. divided hemangiomas, VMs, and lymphatic malformations. VM is subdivided into slow VM and fast arteriovenous malformations [2,3]. VM is observed in 1/5000–10,000 individuals, and approximately 40% of VMs appear in the head and neck regions [4,5,6,7]. Although VM is congenital, it can progress with clinical symptoms with age and does not recede. The progression of VM growth can occur after trauma or puberty [8].

Treatments for VM include embolization, sclerotherapy, surgical removal, and laser treatment, but sclerotherapy has less postoperative scar formation and organic defects and less intraoperative bleeding compared to the other treatments. Thus, it is considered the first choice of treatment. Polidocanol is used for sclerotherapy of esophageal varices and lower limbs.

Among the sclerotherapy agents selected for sclerotherapy, polidocanol is a drug that has fewer side effects and is relatively safer to use than other sclerotherapy agents. Moreover, it has a therapeutic effect on VM. The dosage of polidocanol has not yet been determined. Here, we report the therapeutic effect of polidocanol sclerotherapy in the treatment of VM performed in our department.

## 2. Materials and Methods

A retrospective analysis was performed on patients with VM who underwent percutaneous sclerotherapy with polidocanol between January 2018 and December 2020 at the Department of Oral and Maxillofacial Surgery, Tsukuba University Hospital. This study was conducted in accordance with the Declaration of Helsinki and was approved by the Institutional Review Board of the University of Tsukuba Hospital. Informed consent was waived owing to the retrospective nature of the study (No. R03-161).

Magnetic resonance (MR) images were obtained for a patient clinically diagnosed with VM. However, when the lesion was small or the lesion could not be visualized on MR images due to the influence of the obstruction shadow, the diagnosis was established based on clinical inspection.

**Figure 1:**
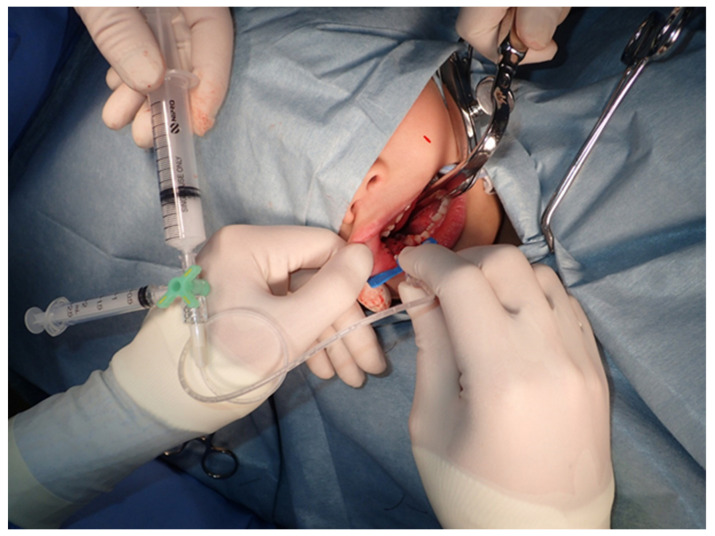
Injecting polidocanol using the foam method.

The size of the VM was measured using MR images or direct oral measurements, and the volume was calculated. The dose of polidocanol administered to patients was 1% (Polidocasklerol^®^, Chemische Fabrik Kreussler & Co. GmbH, Wiesbaden, Germany) to achieve a total dose of ≤2 mg/kg. The foam method [9] was performed using two syringes in the form of a foam with five times the amount of air as polidocanol (Figure 1).

The total dose was 2 mg/kg/times or less and 10 mL or less.

A venous route was secured to prevent allergies, and heart rate, blood pressure, electrocardiogram, and arterial oxygen saturation were monitored. No antibiotics were administered before or after sclerotherapy.

All patients were admitted for observation overnight after sclerotherapy for the first time.

Pain was controlled by administering acetaminophen (300 mg). Pain was assessed using the numerical rating scale (NRS).

Pain evaluation was performed the day after the surgery and 1 week after the surgery.

**Figure 2:**
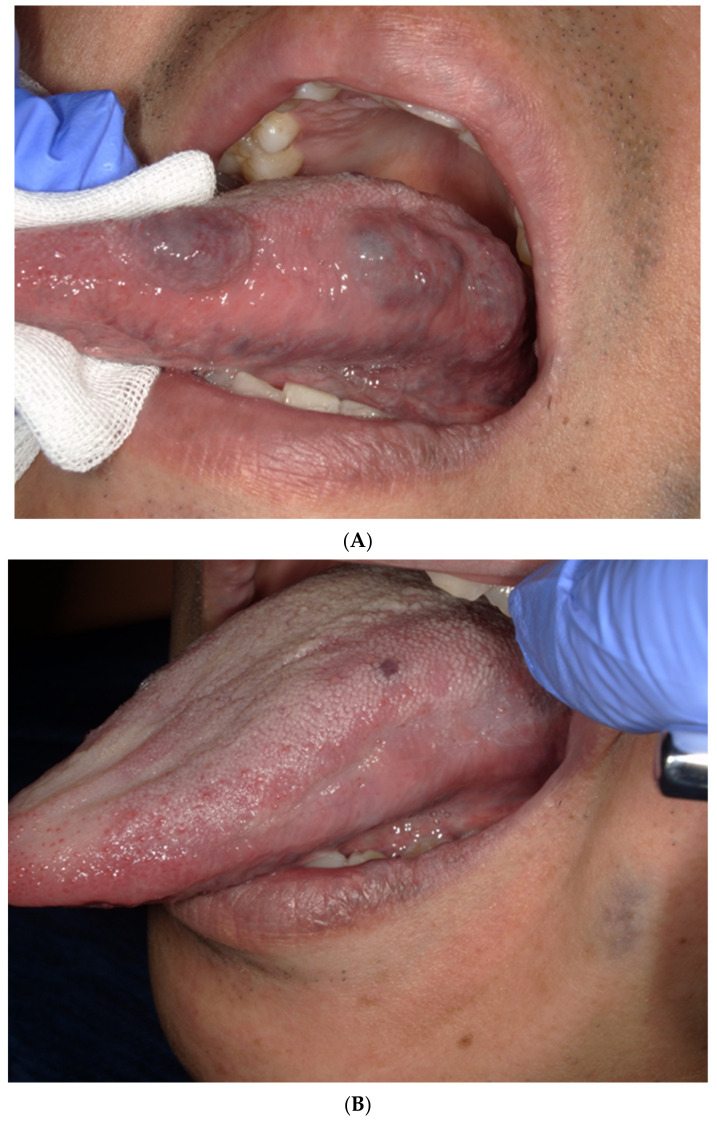
(**A**). The vascular malformation before sclerotherapy. (**B**). An image of a patient with a complete response after sclerotherapy.

Treatment response was evaluated 3 months after the end of the treatment. The evaluation was performed by visual inspection. The VM that completely disappeared was evaluated as a complete response (CR), the size was smaller than that in the initial examination was evaluated as a partial response (PR), the size did not change was evaluated as a stable disease (SD), and the size increased was evaluated as a progressive disease (PD) (Figure 2A,B).

JMP Pro 14 (SAS Institute Inc., Cary, NC, USA) was used for the statistical analysis. Results were considered statistically significant at *p* < 0.05.

## 3. Results

### 3.1. Patient Characteristics

This study comprised nine patients (three men, six women), with 17 sites of VMs. Based on the site, VMs were observed in the tongue, buccal mucosa, lips, and oral floor in eight, three, five, and one patients, respectively. The average size of the site was 3071 mm^3^. The minimum value of the volume was 12 mm^3^, and the maximum value was 14,400 mm^3^. Patient characteristics are summarized in Table 1.

### 3.2. The Treatment and Side Effects

This study comprised 17 sites for oral VMs, with CR, PR, and SD observed at 12, 3, and 2 sites, respectively. The treatment response rate was 15/17 (88%). The two patients who did not respond to sclerotherapy had VM on the floor of the mouth and the tongue, respectively.

No adverse events, such as thrombus, were observed.

The median NRS scores were 2/10 on the next day and 0/10 1 week after the treatment. Most of the pain experienced was self-controlled. Another complication was swelling. Swelling was observed at the site on 15/17 (88%) of patients the day after surgery, but the swelling disappeared in all patients 1 week after the treatment.

The total doses in the response group were 0.5 mL at the minimum and 7 mL at the maximum, with an average of 2.0 mL. The number of administrations was 1 to 3 times, with an average of 1.4 times. The foam method was performed in five patients. Direct injection was performed in 12 patients. The response was not significantly different (*p* = 0.65) in four patients (80%) by the direct injection method.

### 3.3. The Association between the Dose of Polidocanol and the Therapeutic Effect

**Figure 3:**
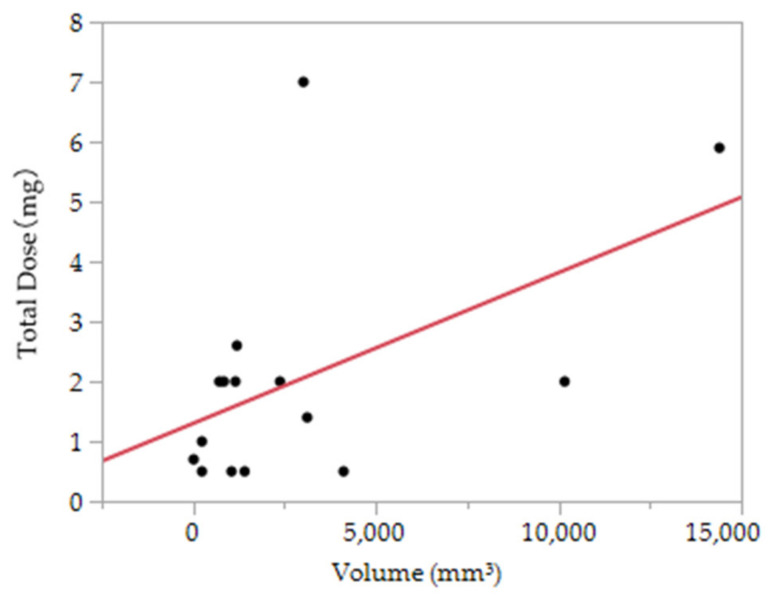
The association between the volume and total dose of polidocanol.

The association between the volume and total dose was examined at the site of successful treatment (Figure 3). From simple regression analysis, the following formula was obtained: 1.30 + 0.00025 × volume (mm3) (mg) (*p* = 0.04).

## 4. Discussion

In recent years, minimally invasive and effective sclerotherapy has been selected for the treatment of VM in the head and neck regions. Dehydrated ethanol, monoethanolamine oleate, and polidocanol are used as curing agents. Dehydrated ethanol causes vascular endothelial damage as soon as it enters the blood vessel with a significantly short time to its onset of effect and causes defect occlusion due to protein denaturation of blood and thrombus and scar formation. Although it is inexpensive and effective, it has some complications, such as tissue necrosis and neuropathy due to extravasation, in addition to pain during drug infusion [10]. Yakes et al. reported 10 side effects of dehydrated ethanol, including minor skin blisters, skin necrosis, transient pain, muscle contractures, sensory nerve injury, motor nerve weakness, superficial cellulitis, deep vein thrombosis, pulmonary embolus, and cardiopulmonary collapse [4]. Monoethanolamine oleate is vascularized by intratumoral injection and develops a chemical reaction on the wall, fibrin, and platelets. The effect is exerted by causing thrombus formation by the precipitation of red blood cells. Although this material has excellent thrombus-forming ability, if it leaks out of the lesion, hemolysis may occur, which may cause acute renal failure. Therefore, it is necessary to inject it together with a contrast medium by digital subtraction angiography so that it does not leak out of the lesion.

In contrast, polidocanol consists of 95% hydroxypolyethoxydodecane and 5% ethyl alcohol. Ethyl alcohol is added as a preservative. Hydroxypolyethoxydodecane is a nonionic surfactant composed of a hydrophobic moiety (dodecyl group) and a hydrophilic moiety (polyoxyethylene group) in the molecule. It is an anionic surfactant that is cytotoxic to endothelial cells and can lyse erythrocytes, leukocytes, and platelets [11,12]. It activates intracellular signaling pathways that regulate intracellular calcium release and nitric oxide production, further inducing cytotoxicity or death. Polidocanol-induced cell death can also result from the activation of the apoptotic pathway or direct chemical toxicity to the cell membrane [13]. In addition, the ionic properties of polidocanol can interfere with plasma and membrane protein synthesis. Polidocanol also has an antiangiogenesis effect [14]. It causes hemolysis and fibrotic tissue damage over time. Polidocanol is used in the treatment of varicose veins in addition to sclerotherapy for hemangiomas by utilizing its action [15]. There are also reports showing the effectiveness of polidocanol sclerotherapy for mucocele of the minor salivary gland [16].

The curative effect of polidocanol is inferior to that of ethanol. Ethanol sclerotherapy reportedly reduces or improves lesions in 64–96% of cases [17,18,19,20,21]. In the present study, CR, PR, and SD were observed in 12, 3, and 2 sites, respectively. The treatment response rate was 88%. Polidocanol sclerotherapy has the same effect as ethanol with fewer side effects.

Mimura et al. performed sclerotherapy using 3% polidocanol under general anesthesia and fluoroscopy in 16 patients with VM in the limbs, head, and neck and injected a total of 40–630 mg. It was reported that 13 out of 14 patients who could be followed up were able to obtain therapeutic effects, such as the disappearance of lesions [22]. Miura et al. also reported that 29 patients with VM with pain underwent sclerotherapy using polidocanol, 12 were in remission, 14 were improved, 2 were unchanged, and 1 worsened [23]. Hou et al. reported that sclerotherapy in children had a therapeutic effect on the head and neck in 12/16 cases (75%) [24].

Qiu et al. reported that in 163 patients who underwent sclerotherapy for VM, blood pressure decreased, and bradycardia was observed in 0.61% of patients, but it was clinically difficult to distinguish them from the vagal reflex [25]. Marrocco-Trischitta et al. [26], Shimo et al. [27], and others have reported cases of cardiac arrest in children, and the drugs administered at this time were 1% polidocanol (4 mL) (body weight 20 kg) and 3% polidocanol (10 mL) (body weight 15.6 kg). No serious side effects, such as skin necrosis, were observed in all patients. We did not find any serious side effects other than pain or swelling of the injected site. The median pain score based on the NRS was 2/10, and swelling was observed at the site in 88% of patients the day after surgery.

A simple regression analysis provided the formula 1.30 + 0.00025 × volume (mm^3^) to assess the association between successful cases and a 1% polidocanol dose. The guideline for the required 1% polidocanol dose derived from this formula was 1.55 mL for 1 cm^3^ hemangiomas and 3.3 mL for 2 cm^3^ hemangiomas. When converted to a body weight of 60 kg, the total dose was 12 mg, and up to 1% polidocanol (12 mL) was administered. When there are many sites, it is possible to obtain a therapeutic effect using 2 mg (2 mL) if the size is approximately 1000 mm^3^. The problem with this statistical method is simply the correlation between volume and dose, not considering other factors. The number of cases was too small for this study. The dose study reported the administration of 9–450 mg (average 156 mg) for 22−243 mm (average 92 mm) hemangiomas [22]. This is a study for systemic hemangiomas, so the size is large and the volume is large. This has not been considered. For large hemangiomas, it is considered effective to use the foam method, but in this study, there were many smaller hemangiomas than other reports, and the effectiveness of the foam method could not be examined.

In 2003, Cabrera et al. [28] reported foam sclerotherapy with 0.25–4% polidocanol and carbon dioxide, which was effective in 92% of patients, with VMs completely disappearing in 36% of patients. Yamaki et al. [29] conducted a randomized controlled trial of liquid sclerotherapy and foam sclerotherapy with 1% polidocanol and 10% ethanolamine oleate. According to their study, foam sclerotherapy was significantly more effective than liquid sclerotherapy (90% vs. 63%, *p* = 0.002), and the amount of hardener used was significantly smaller. In this study, the results were almost the same for the administration, foam, and direct injection methods, although the number of patients was small. In fact, with the foam method, it is difficult to inject a small dose of polidocanol in the oral cavity. Considering that the foam method is an effective method for large VMs, calculating the effective dose by volume and using the foam method together in consideration of the total dose are considered clinically beneficial.

This study was a single-center study with a limited number of cases. We would like to increase the number of cases and consider research at other facilities. In addition, it is necessary to verify the validity of the results obtained in this study in a prospective study in the future. This study made it difficult to quantify intraoral measurements. In particular, it was difficult to evaluate small lesions that could not be visualized by MRI. Measurement using echo and its quantification will be an issue.

## 5. Conclusions

Polidocanol sclerotherapy is an effective treatment method for oral VM. The formula for calculating the required amount of polidocanol was 1.3 + 0.00025 × volume (mm^3^) (mg). It may be a reference for the amount to be administered according to the volume.

## Figures and Tables

**Table 1:** Summary of study patients.

Case	Age (Years)	Sex	Height (cm)	Body Weight (Kg)	Primary	Volume (mm^3^)	Total Dose (mL)	Administration Method	Number of Administrations	NRS Score the Next Day	NRS Score after 1 Week	Effect Mesurement
1	5	F	113	20	Tongue	3016	7	Foam	3	4	0	PR
2	42	M	164	62	Right tongue	1408	0.5	Foam	1	6	0	CR
3	42	M	164	62	Left tongue	1050	0.5	Foam	1	6	0	CR
4	42	M	164	62	Buccal mucosa	4116	0.5	Foam	1	6	0	CR
5	44	F	161	61	Oral floor	2912	4.5	Foam	2	5	0	SD
6	44	F	161	61	Tongue	1800	1	Local injection	1	3	1	SD
7	44	F	161	61	Lip	240	0.5	Local injection	1	0	0	CR
8	23	F	157	47	Lip	240	1	Local injection	1	5	0	CR
9	23	F	157	47	Tongue	10,166	2	Local injection	2	-	-	PR
10	81	F	162	61	Tongue	14,400	5.9	Local injection	2	2	0	PR
11	81	F	162	61	Lip	1197	2.6	Local injection	2	0	0	CR
12	81	M	153	49	Tongue	840	2	Local injection	1	0	0	CR
13	41	M	165	80	Tongue	1155	2	Local injection	1	0	0	CR
14	41	M	165	80	Tongue	2376	2	Local injection	1	0	0	CR
15	41	M	165	80	Buccal mucosa	3120	1.4	Local injection	1	0	0	CR
16	83	F	152	44	Lip	715	2	Foam	1	0	0	CR
17	75	F	149	49	ip	12	0.7	Local injection	1	1	0	CR

NRS, numerical rating scale; CR, complete response; PR, partial response; SD, stable disease.

## Data Availability

Data are available upon reasonable request.
